# Factors Associated with the Practice of Assessing Drug–Drug Interactions Among Pharmacists in Saudi Arabia

**DOI:** 10.3390/healthcare12222285

**Published:** 2024-11-15

**Authors:** Khalid Alhussain, Abdullah Al Dandan, Haider Al Elaiwi, Hassan Al Wabari, Ali Al Abdulathim, Sulaiman Almohaish

**Affiliations:** 1 Department of Pharmacy Practice, College of Clinical Pharmacy, King Faisal University, Al Ahsa 31982, Saudi Arabia; salmohaish@kfu.edu.sa; 2 Al-Dawaa Pharmacy, Al Ahsa 31982, Saudi Arabia; abdulah.aldandan@gmail.com (A.A.D.); haideralelaiwi@gmail.com (H.A.E.); hassn-al-wabari@hotmail.com (H.A.W.); araalabdulathim@gmail.com (A.A.A.)

**Keywords:** drug–drug interactions, pharmacists’ knowledge, pharmacists’ attitude, pharmacy practice, patient safety

## Abstract

Background: Drug–drug interactions (DDIs) occur when two or more drugs are administered concomitantly, changing the pharmacokinetics or pharmacodynamics of a drug’s characteristics. Despite the advances in health technology, DDIs remain a concern to patient safety. This study aimed to (1) assess the knowledge, attitude, and practice of hospital and community pharmacists toward DDIs in Saudi Arabia and (2) examine factors associated with their practice. Methods: A cross-sectional study was conducted using an online self-administered questionnaire targeting hospital and community pharmacists working in Saudi Arabia. The study questionnaire consisted of five sections: demographics, knowledge, attitude, and practice toward DDIs, as well as pharmacy characteristics. Descriptive statistics were used to summarize the characteristics of participants as count and percentage. Chi-square tests were used to examine associations between practice variables and other independent variables. Results: A total of 131 pharmacists participated in the study. The majority were males (81.7%), aged 26–35 years (64.9%), and worked in community pharmacies (81.7%). Nearly half of the participants reported optimal practice regarding checking drug interactions before dispensing any drug. Factors associated with the practice of checking DDIs were found to be gender, perceived workload, perceived knowledge, and attitude variables. Regarding the practice of asking patients about their prescription and OTC drugs, there were statistically significant differences between hospital and community pharmacists. Conclusions: Our findings on both community and hospital pharmacists in Saudi Arabia reveal that pharmacists’ attitudes and perceived knowledge might influence the practice of pharmacists toward DDIs.

## 1. Introduction

Administering two or more drugs simultaneously may lead to drug–drug interactions (DDIs), where either the pharmacokinetics or pharmacodynamics of a drug may change [1,2]. DDIs might produce antagonistic or synergistic effects, both of which may result in drug toxicity or undesirable therapeutic effects with adverse patient outcomes [3]. The seriousness of these adverse outcomes varies greatly from mild to severe symptoms. For instance, DDIs can result in emergency room visits and hospitalization [2,4]. DDIs remain a healthcare concern worldwide despite enormous breakthroughs and advances in health technology. A recent systematic review and meta-analysis assessing the prevalence of DDIs in inpatient settings revealed that the pooled prevalence of potential DDIs was 64.9%, and it was 17.2% for clinically evident DDIs [5]. The prevalence of DDIs is even higher among elderly patients; it was estimated to range from 0.8% to 90.6% across studies [6]. For example, a study conducted in Saudi Arabia found that the overall prevalence of DDIs among the elderly at an ambulatory care pharmacy was 90.6% [7]. Another study has indicated that about one in five patients exposed to a potential DDI might experience adverse drug reactions [8]. This percentage could increase as the number of concomitantly administered drugs increases. Moreover, DDIs accounted for around 7% of all hospital admissions caused by drug-related problems in Saudi Arabia [9].

Given the high prevalence of DDIs, their negative consequences will be more significant. DDIs are one of the causes of adverse drug reactions [8]. A study among elderly patients revealed that the incidence of adverse drug reactions caused by DDIs was 6% in outpatient settings [10]. Such adverse drug reactions may lead to hospitalization, as previous research has shown [11,12]. For example, one study found that about 10% of adverse drug reactions caused by DDIs in patients with cardiovascular diseases (e.g., heart rate and rhythm abnormalities or bleeding) resulted in hospital admissions [12]. Furthermore, DDIs occurring during hospitalization may increase the length of stay [13]. In addition to the health burden, DDIs have a substantial economic burden. Several studies have documented the increased cost associated with DDIs among different populations [14,15,16]. 

To minimize DDIs and their adverse outcomes, pharmacists can play a significant role as they have knowledge about drugs and are trained to ensure medication safety [17,18]. For example, pharmacists can perform medication reconciliation, where they review all prescription medications and over-the-counter (OTC) drugs taken by a patient to avoid any possible DDIs [19]. However, several challenges may hinder pharmacists from effectively assessing potential DDIs in hospital or community pharmacies, such as inadequate knowledge, workplace pressures, and lack of drug interaction checkers [20]. Due to these, previous studies have assessed pharmacists’ knowledge regarding DDIs in Saudi Arabia [21,22,23]. The results from these studies indicate that the pharmacists’ knowledge of drug interactions was inadequate. These studies only included community pharmacists and were limited to specific regions (the central region and Jeddah). To our knowledge, only one study evaluated pharmacists’ attitudes and practices toward DDIs [21], but it did not investigate factors related to pharmacists’ practice toward DDIs. Therefore, the current study aimed to (1) assess the knowledge, attitude, and practice of hospital and community pharmacists toward DDIs in Saudi Arabia and (2) examine factors associated with pharmacists’ practice. 

## 2. Materials and Methods

### 2.1. Study Design and Sample

A cross-sectional study was conducted using an anonymous online self-administered questionnaire. The questionnaire was carried out in 2023 from March to May using Google Forms. This study targeted hospital and community pharmacists in both the private and public sectors in Saudi Arabia.

### 2.2. Study Questionnaire

The study questionnaire consisted of five sections (see Supplementary Materials for details). The first section was about demographic data (e.g., age, gender, and education). The second section assessed pharmacists’ knowledge about DDIs. The knowledge questions consisted of 16 drug pairs; these pairs were selected based on their prevalence in the literature and drug availability in Saudi Arabia based on the Saudi Food and Drug Administration (SFDA). Pharmacists were asked to classify each drug pair into one of the following: “contraindication”, “may be used together with monitoring”, “no interaction”, or “not sure”. Each question has one correct answer, which is worth 1 point. This means the maximum knowledge score is 16. Knowledge scores ranged from 0 to 16 and were interpreted as follows: poor (0–5.33), good (5.34–10.67), and excellent (10.68–16). The third section was about the attitude of pharmacists toward DDIs. It includes three statements, and the pharmacists were asked to provide their level of agreement on a scale ranging from strongly disagree to strongly agree. Participants responding with “strongly disagree”, “disagree”, or “neither disagree nor agree” were classified as having negative attitudes. On the other hand, those responding with “agree” or “strongly agree” were classified as having positive attitudes. The fourth section assessed pharmacists’ practice toward DDIs. For example, participants were asked the following question: “In general, before dispensing any drug (new or refill), how often do you consider its potential interactions?”, and the response scale ranged from never to always. Participants responding to the practice questions with “never”, “rarely”, or “sometimes” were classified as having suboptimal practice, while those responding with “often” or “always” were classified as having optimal practice. The fifth section was about pharmacy characteristics (e.g., perceived workload, number of staff, and availability of drug information resources). 

### 2.3. Ethical Approval

Ethical approval was obtained from the Research Ethics Committee at King Faisal University, Saudi Arabia (KFU-REC-2023-MAR-ETHICS673).

### 2.4. Statistical Analyses

Descriptive statistics were used to summarize the characteristics of the study participants as count and percentage. Chi-square tests were used to examine associations between practice variables and other independent variables. All analyses were performed using IBM SPSS software Version 25.0.

## 3. Results

### 3.1. Characteristics of the Study Sample

The characteristics of the study sample (n = 131) are presented in Table 1. The majority of participants were males (81.7%) and around 26–35 years of age (64.9%). Most participants had a bachelor’s/PharmD degree (90.8%), were working in community pharmacies (81.7%), and had more than five years of experience (58.0%). About 45.0% of the participants were Saudis, and 30.5% were from the Eastern region. 

### 3.2. Pharmacists’ Knowledge, Attitude, and Practice Toward DDIs

Table 2 summarizes the level of pharmacists’ knowledge, attitude, and practice toward DDIs. In our sample, 25.2% of pharmacists perceived that they had very good/excellent knowledge about DDIs. When knowledge was measured, only 12.2% had excellent knowledge about DDIs. The majority of the participants had positive attitudes toward checking for drug–drug interactions. In terms of practice variables, nearly half of the participants reported optimal practice regarding checking drug interactions before dispensing any drug. About 67.2% stated that they often/always asked patients about their prescription drugs, and 62.6% often/always asked about over-the-counter drugs. There were statistically significant differences between hospital and community pharmacists in terms of asking patients about their drugs. It was found that 72.0% of community pharmacists reported optimal practice regarding asking patients about their prescription drugs compared to 47.8% of hospital pharmacists (*p* = 0.025). Regarding the practice of asking patients about their OTC drugs, we observed that 70.1% of community pharmacists reported optimal practice, while 30.4% of hospital pharmacists reported optimal practice (*p* < 0.001).

### 3.3. Factors Associated with Pharmacists’ Practice Toward DDIs

#### 3.3.1. Practice of Checking Drug–Drug Interactions

Statistically significant associations were observed between gender, perceived workload, perceived knowledge, and attitude variables and the practice of checking DDIs (see Table 3A). A higher proportion of male pharmacists reported optimal practice of checking DDIs than female pharmacists (55.1% vs. 25.0%). We also found that 57.9% of pharmacists reporting a high/very high workload stated that they checked for DDIs, whereas 18.2% of those reporting low/very low workload checked for DDIs in practice. Also, pharmacists who believed that they had very good/excellent knowledge about DDIs were more involved in checking for DDIs compared to those who believed that their knowledge about DDIs was poor/fair (69.7% vs. 25.0%).

#### 3.3.2. Practice of Asking Patients About Their Prescription Drugs

There were statistically significant associations of nationality, number of pharmacists in a shift, and measured knowledge with the practice of asking patients about their prescription drugs (see Table 3B). We found that 87.5% of pharmacists with excellent measured knowledge reported optimal practice regarding asking patients about their prescription drugs, while 53.3% of those having poor measured knowledge reported optimal practice. The percentage of non-Saudi pharmacists asking for prescription drugs was higher than their Saudi counterparts (75.0% vs. 57.6%).

#### 3.3.3. Practice of Asking Patients About OTC Drugs

There were statistically significant associations of nationality, graduation country, and region with the practice of asking patients about OTC drugs (See Table 3C). A higher percentage of non-Saudi pharmacists reported that they asked patients about their OTC drugs compared to Saudi pharmacists (72.2% vs. 50.8%). We also found that 47.5% of pharmacists working in the Eastern region stated that they asked patients about their OTC drugs, whereas 69.2% of those working in other regions reported optimal practice of asking about OTC drugs.

## 4. Discussion

Our study is the first to shed light on the knowledge, attitude, and practice of pharmacists regarding DDIs with insights into factors influencing their practice in all major regions in Saudi Arabia. In this study, we were able to reveal a notable gap in pharmacists’ knowledge concerning DDIs, with only a small percentage (12.2%) demonstrating excellent knowledge. Based on this finding, the previous knowledge assessment and results that were conducted in small parts of Saudi Arabia can be generalized as our study covered hospitals and community pharmacy centers in the three major regions of Saudi Arabia [21,22,23]. Such a deficit in knowledge poses a great threat to patient safety, as pharmacists may underestimate their lack of knowledge about DDIs and the negative consequences associated with it, which may lead to worse outcomes. When we examined the relationship between pharmacists’ knowledge about DDIs and their practice, we found that measured knowledge was not significantly associated with the optimal practice regarding checking DDIs. This knowledge–practice gap could be due to the fact that some pharmacists rely on computer programs to detect DDIs before dispensing any drug. However, these programs have some limitations (e.g., technical issues) and may create more dependent pharmacists who do not feel the urge to improve. Further, knowledge about DDIs can vary based on the practice site or the specialty of the pharmacists. For example, some DDIs are encountered more in community practices than in hospital settings and vice versa. In terms of practice regarding asking patients about their prescriptions, measured knowledge was found to be associated with optimal practice. 

Despite limited knowledge, the majority of pharmacists exhibited a positive attitude toward checking for DDIs. This suggests a willingness among pharmacists to address this issue, which is encouraging for interventions aimed at improving DDI management. However, there remains a disparity between attitude and practice, as evidenced by the relatively lower proportion, around 67.2%, of pharmacists who consistently consider asking about home medication or patients’ over-the-counter drug use before dispensing medications. This disconnection underscores the need for targeted strategies to bridge the gap between attitude and practice, such as providing scientific training programs or activities that change work cultures at these sites.

In our study, we tried to look for reasons behind our findings, and we found several factors that influence pharmacists’ practice regarding DDIs. Workload and perceived knowledge emerged as significant determinants, and higher levels of knowledge and workload were associated with better practice toward DDI checking and management. This highlights the importance of addressing individual and organizational factors in promoting good clinical practice and the significance of the level of knowledge when dealing with DDIs.

The high prevalence of DDIs, coupled with the suboptimal knowledge and practice observed among pharmacists, highlight the urgent need for interventions to enhance the practice and attitude toward DDIs potential to harm and ways of prevention in Saudi Arabia [7,8,10,11]. Given the potential for DDIs to result in serious adverse drug events and subsequent hospitalization, addressing this issue is paramount to ensuring patient safety and optimizing healthcare outcomes [2,4]. Pharmacists, who are considered frontline healthcare providers, play a major role in identifying and managing DDIs, thereby safeguarding patient well-being. In order for pharmacists to fulfill their obligations to their patients and the community, regular continuing medical education (CME) programs are essential for improving their knowledge and quality of healthcare services [24]. In 1962, a medical education meeting of the College of General Practice of Canada concluded that continuing CME is a crucial component of modern medicine since it preserves the healthcare providers’ capacity to deliver high-quality patient care [25]. The Accreditation Council for Pharmacy Education’s CE (Continuing Education) is a structured educational program that helps pharmacists maintain and enhance their professional development to deliver the highest caliber of services [26]. To summarize, continuing medical education and development keeps healthcare professionals capable of delivering the best quality patient care and is a crucial part of contemporary medical practice.

While our study provides important insights into the knowledge, attitude, and practice of pharmacists regarding DDIs in Saudi Arabia, there are some limitations that should be considered. Its cross-sectional design gives an idea about the problem, but survey data may be subject to recall bias. Investigating self-reported knowledge and skills can be affected by self-perception bias, where respondents may tend to overestimate or underestimate their knowledge or skills, especially if they lack adequate experience or if they feel that they have substantial gaps in their knowledge. Respondents may also differ in how they interpret their knowledge and skills. Further, cultural differences play a critical role in how individuals reflect on themselves, as some cultures place a higher value on humility, while others tend to foster greater self-esteem. Future research could focus on longitudinal healthcare quality-oriented projects to gain a deeper understanding of the factors influencing pharmacists’ behavior regarding DDIs.

Finally, this study highlights the importance of addressing the knowledge-practice gap among both community and hospital pharmacists regarding DDIs in all regions of Saudi Arabia. Enhancing pharmacists’ awareness, attitudes, and practices concerning DDIs can mitigate the risks associated with DDIs and improve patient safety. We recommend conducting future quality-oriented projects between policymakers, educators, and healthcare practitioners to implement effective interventions and ensure optimal practices across all healthcare settings.

## 5. Conclusions

The present study shows that about half of the pharmacists reported suboptimal practice regarding checking drug–drug interactions. This may lead to poor health outcomes and negatively affect patient safety. Therefore, interventions improving pharmacists’ practice concerning DDIs should be developed and implemented. These interventions should consider factors associated with pharmacists’ practice toward DDIs, such as gender, perceived workload, perceived knowledge, and attitude variables. In addition, future quality-oriented projects involving policymakers, educators, and healthcare practitioners should be conducted.

## Figures and Tables

**Table 1:** Characteristics of study sample (n = 131).

Variables	N	%
**Age**		
22–25 years old	20	15.3%
26–35 years old	85	64.9%
Above 35 years old	26	19.8%
**Gender**		
Male	107	81.7%
Female	24	18.3%
**Nationality**		
Saudi	59	45.0%
Non-Saudi	72	55.0%
**Marital status**		
Married	84	64.1%
Unmarried	47	35.9%
**Education**		
Diploma	8	6.1%
Bachelor’s degree or Pharm D	119	90.8%
Master’s degree	4	3.1%
**Time of graduation**		
This year	9	6.9%
1–5 years ago	51	38.9%
More than 5 years ago	71	54.2%
**Country of graduation**		
Saudi Arabia	59	45.0%
Other	72	55.0%
**Workplace**		
Hospital pharmacy	23	17.6%
Community pharmacy	107	81.7%
**Working hours**		
8 h/day or less	116	88.5%
12 h/day or more	15	11.5%
**Years of practice**		
Less than a year	17	13.0%
1–5 years	38	29.0%
More than 5 years	76	58.0%
**Type of working institution**		
Private	105	80.2%
Public	26	19.8%
**Perceived workload**		
Very low/low	11	8.4%
Moderate	44	33.6%
High/very high	76	58.0%
**Number of other pharmacists in a shift**		
None	24	18.3%
1–2	62	47.3%
3 or more	45	34.4%
**Number of technicians in a shift**		
None	57	43.5%
1–2	52	39.7%
3 or more	22	16.8%
**Number of interns in a shift**		
None	74	56.5%
1–2	34	26.0%
3 or more	23	17.6%
**Availability of drug–drug interaction checker**		
Yes	74	56.5%
No	57	43.5%
**Region**		
Eastern	40	30.5%
Other	91	69.5%

Note: There were missing data for workplace variable.

**Table 2:** Level of pharmacists’ knowledge, attitude, and practice toward DDIs by workplace.

	Total Sample		Hospital Pharmacists		Community Pharmacists		*p*-Value
	N	%	N	%	N	%	
All	131	100.0	23	17.6	107	81.7	
**Knowledge variables**							
**Perceived knowledge**							0.282
Poor/fair	20	15.3%	2	8.7%	18	16.8%	
Good	78	59.5%	17	73.9%	60	56.1%	
Very good/excellent	33	25.2%	4	17.4%	29	27.1%	
**Measured knowledge**							0.535
Poor	45	34.4%	10	43.5%	34	31.8%	
Good	70	53.4%	11	47.8%	59	55.1%	
Excellent	16	12.2%	2	8.7%	14	13.1%	
**Attitude variables**							
**Some drug–drug interactions can be fatal**							0.375
Negative attitude	33	25.2%	4	17.4%	28	26.2%	
Positive attitude	98	74.8%	19	82.6%	79	73.8%	
**Pharmacist should update their knowledge about drug–drug interactions**							0.423
Negative attitude	32	24.4%	4	17.4%	27	25.2%	
Positive attitude	99	75.6%	19	82.6%	80	74.8%	
**Pharmacist should check for drug–drug interactions**							0.423
Negative attitude	32	24.4%	4	17.4%	27	25.2%	
Positive attitude	99	75.6%	19	82.6%	80	74.8%	
**Practice variables**							
**In general, before dispensing any drug (new or refill), how often do you consider its potential interactions?**							0.818
Suboptimal practice	66	50.4%	12	52.2%	53	49.5%	
Optimal practice	65	49.6%	11	47.8%	54	50.5%	
How often do you ask your patients about their prescription drugs? *							0.025
Suboptimal practice	43	32.8%	12	52.2%	30	28.0%	
Optimal practice	88	67.2%	11	47.8%	77	72.0%	
How often do you ask your patients about their over-the-counter (OTC) drugs? *							<0.001
Suboptimal practice	49	37.4%	16	69.6%	32	29.9%	
Optimal practice	82	62.6%	7	30.4%	75	70.1%	
**How often do you come across drug–drug interactions?**							0.925
Suboptimal practice	79	60.3%	14	60.9%	64	59.8%	
Optimal practice	52	39.7%	9	39.1%	43	40.2%	

Note: * indicates statistically significant results (*p*-value < 0.05).

**Table 3:** A. Characteristics of the study sample by their practice of checking drug–drug interactions. B. Characteristics of the study sample by their practice of asking patients about prescription drugs. C. Characteristics of the study sample by their practice of asking patients about over-the-counter (OTC) drugs.

A					
	Optimal Practice Regarding Checking Drug–Drug Interactions		Suboptimal Practice Regarding Checking Drug–Drug Interactions		*p*-Value
	N	%	N	%	
**Age groups**					0.392
22–25 years old	9	45.0%	11	55.0%	
26–35 years old	40	47.1%	45	52.9%	
Above 35 years old	16	61.5%	10	38.5%	
Gender *					0.008
Male	59	55.1%	48	44.9%	
Female	6	25.0%	18	75.0%	
**Nationality**					0.133
Saudi	25	42.4%	34	57.6%	
Non-Saudi	40	55.6%	32	44.4%	
**Marital status**					0.805
Married	41	48.8%	43	51.2%	
Unmarried	24	51.1%	23	48.9%	
**Graduation year**					0.936
1–5 years ago	30	50.0%	30	50.0%	
More than 5 years ago	35	49.3%	36	50.7%	
**Graduation country**					0.250
Saudi Arabia	26	44.1%	33	55.9%	
Other	39	54.2%	33	45.8%	
**Working hours**					0.760
8 h/day or less	57	49.1%	59	50.9%	
12 h/day or more	8	53.3%	7	46.7%	
**Years of practice**					0.925
Less than a year	9	52.9%	8	47.1%	
1–5 years	18	47.4%	20	52.6%	
More than 5 years	38	50.0%	38	50.0%	
**Type of working institution**					0.693
Private	53	50.5%	52	49.5%	
Public	12	46.2%	14	53.8%	
Perceived workload *					0.028
Very low/low	2	18.2%	9	81.8%	
Moderate	19	43.2%	25	56.8%	
High/very high	44	57.9%	32	42.1%	
**Number of other pharmacists in a shift**					0.187
None	15	62.5%	9	37.5%	
1–2	32	51.6%	30	48.4%	
3 or more	18	40.0%	27	60.0%	
**Number of technicians in a shift**					0.348
None	30	52.6%	27	47.4%	
1–2	22	42.3%	30	57.7%	
3 or more	13	59.1%	9	40.9%	
**Number of interns in a shift**					0.135
None	37	50.0%	37	50.0%	
1–2	13	38.2%	21	61.8%	
3 or more	15	65.2%	8	34.8%	
**Availability of drug–drug interaction checker**					0.247
Yes	40	54.1%	34	45.9%	
No	25	43.9%	32	56.1%	
**Region**					0.954
Eastern region	20	50.0%	20	50.0%	
Others	45	49.5%	46	50.5%	
Perceived knowledge *					0.006
Poor/fair	5	25.0%	15	75.0%	
Good	37	47.4%	41	52.6%	
Very good/excellent	23	69.7%	10	30.3%	
**Measured knowledge**					0.146
Poor	17	37.8%	28	62.2%	
Good	39	55.7%	31	44.3%	
Excellent	9	56.3%	7	43.8%	
Some drug–drug interactions can be fatal *					0.010
Negative attitude	10	30.3%	23	69.7%	
Positive attitude	55	56.1%	43	43.9%	
Pharmacist should update their knowledge about drug–drug interactions *					0.005
Negative attitude	9	28.1%	23	71.9%	
Positive attitude	56	56.6%	43	43.4%	
Pharmacist should check for drug–drug interactions *					0.005
Negative attitude	9	28.1%	23	71.9%	
Positive attitude	56	56.6%	43	43.4%	
**B**					
	**Optimal Practice Regarding Asking About Prescription Drugs**		**Suboptimal Practice Regarding Asking About Prescription Drugs**		***p*** **-Value**
	**N**	**%**	**N**	**%**	
**Age groups**					0.772
22–25 years old	13	65.0%	7	35.0%	
26–35 years old	56	65.9%	29	34.1%	
Above 35 years old	19	73.1%	7	26.9%	
**Gender**					0.589
Male	73	68.2%	34	31.8%	
Female	15	62.5%	9	37.5%	
Nationality *					0.035
Saudi	34	57.6%	25	42.4%	
Non-Saudi	54	75.0%	18	25.0%	
**Marital status**					0.184
Married	53	63.1%	31	36.9%	
Unmarried	35	74.5%	12	25.5%	
**Graduation year**					0.389
1–5 years ago	38	63.3%	22	36.7%	
More than 5 years ago	50	70.4%	21	29.6%	
**Graduation country**					0.174
Saudi Arabia	36	61.0%	23	39.0%	
Other	52	72.2%	20	27.8%	
**Working hours**					0.589
8 h/day or less	77	66.4%	39	33.6%	
12 h/day or more	11	73.3%	4	26.7%	
**Years of practice**					0.935
Less than a year	11	64.7%	6	35.3%	
1–5 years	25	65.8%	13	34.2%	
More than 5 years	52	68.4%	24	31.6%	
**Type of working institution**					0.828
Private	71	67.6%	34	32.4%	
Public	17	65.4%	9	34.6%	
**Perceived workload**					0.056
Very low/low	5	45.5%	6	54.5%	
Moderate	26	59.1%	18	40.9%	
High/very high	57	75.0%	19	25.0%	
Number of other pharmacists in a shift *					0.046
None	17	70.8%	7	29.2%	
1–2	47	75.8%	15	24.2%	
3 or more	24	53.3%	21	46.7%	
**Number of technicians in a shift**					0.642
None	40	70.2%	17	29.8%	
1–2	35	67.3%	17	32.7%	
3 or more	13	59.1%	9	40.9%	
**Number of interns in a shift**					0.741
None	50	67.6%	24	32.4%	
1–2	24	70.6%	10	29.4%	
3 or more	14	60.9%	9	39.1%	
**Availability of drug–drug interaction checker**					0.521
Yes	48	64.9%	26	35.1%	
No	40	70.2%	17	29.8%	
**Region**					0.246
Eastern region	24	60.0%	16	40.0%	
Others	64	70.3%	27	29.7%	
**Perceived knowledge**					0.176
Poor/fair	11	55.0%	9	45.0%	
Good	51	65.4%	27	34.6%	
Very good/excellent	26	78.8%	7	21.2%	
Measured knowledge *					0.024
Poor	24	53.3%	21	46.7%	
Good	50	71.4%	20	28.6%	
Excellent	14	87.5%	2	12.5%	
**Some drug–drug interactions can be fatal**					0.353
Negative attitude	20	60.6%	13	39.4%	
Positive attitude	68	69.4%	30	30.6%	
**Pharmacist should update their knowledge about drug–drug interactions**					0.130
Negative attitude	18	56.3%	14	43.8%	
Positive attitude	70	70.7%	29	29.3%	
**Pharmacist should check for drug–drug interactions**					0.130
Negative attitude	18	56.3%	14	43.8%	
Positive attitude	70	70.7%	29	29.3%	
**C**					
	**Optimal Practice Regarding Asking About OTC Drugs**		**Suboptimal Practice Regarding Asking About OTC Drugs**		***p*** **-Value**
	**N**	**%**	**N**	**%**	
**Age groups**					0.734
22–25 years old	12	60.0%	8	40.0%	
26–35 years old	52	61.2%	33	38.8%	
Above 35 years old	18	69.2%	8	30.8%	
**Gender**					0.345
Male	69	64.5%	38	35.5%	
Female	13	54.2%	11	45.8%	
Nationality *					0.012
Saudi	30	50.8%	29	49.2%	
Non-Saudi	52	72.2%	20	27.8%	
**Marital status**					0.827
Married	52	61.9%	32	38.1%	
Unmarried	30	63.8%	17	36.2%	
**Graduation year**					0.197
1–5 years ago	34	56.7%	26	43.3%	
More than 5 years ago	48	67.6%	23	32.4%	
Graduation country *					0.031
Saudi Arabia	31	52.5%	28	47.5%	
Other	51	70.8%	21	29.2%	
**Working hours**					0.825
8 h/day or less	73	62.9%	43	37.1%	
12 h/day or more	9	60.0%	6	40.0%	
**Years of practice**					0.441
Less than a year	10	58.8%	7	41.2%	
1–5 years	21	55.3%	17	44.7%	
More than 5 years	51	67.1%	25	32.9%	
**Type of working institution**					0.138
Private	69	65.7%	36	34.3%	
Public	13	50.0%	13	50.0%	
**Perceived workload**					0.211
Very low/low	5	45.5%	6	54.5%	
Moderate	25	56.8%	19	43.2%	
High/very high	52	68.4%	24	31.6%	
**Number of other pharmacists in a shift**					0.304
None	13	54.2%	11	45.8%	
1–2	43	69.4%	19	30.6%	
3 or more	26	57.8%	19	42.2%	
**Number of technicians in a shift**					0.442
None	39	68.4%	18	31.6%	
1–2	31	59.6%	21	40.4%	
3 or more	12	54.5%	10	45.5%	
**Number of interns in a shift**					0.959
None	46	62.2%	28	37.8%	
1–2	21	61.8%	13	38.2%	
3 or more	15	65.2%	8	34.8%	
**Availability of drug–drug interaction checker**					0.907
Yes	46	62.2%	28	37.8%	
No	36	63.2%	21	36.8%	
Region*					0.018
Eastern region	19	47.5%	21	52.5%	
Others	63	69.2%	28	30.8%	
**Perceived knowledge**					0.538
Poor/fair	11	55.0%	9	45.0%	
Good	48	61.5%	30	38.5%	
Very good/excellent	23	69.7%	10	30.3%	
**Measured knowledge**					0.124
Poor	23	51.1%	22	48.9%	
Good	49	70.0%	21	30.0%	
Excellent	10	62.5%	6	37.5%	
**Some drug–drug interactions can be fatal**					0.785
Negative attitude	20	60.6%	13	39.4%	
Positive attitude	62	63.3%	36	36.7%	
**Pharmacist should update their knowledge about drug–drug interactions**					0.990
Negative attitude	20	62.5%	12	37.5%	
Positive attitude	62	62.6%	37	37.4%	
**Pharmacist should check for drug–drug interactions**					0.990
Negative attitude	20	62.5%	12	37.5%	
Positive attitude	62	62.6%	37	37.4%	

Note: * indicates statistically significant results (*p*-value < 0.05).

## Data Availability

The data used to support the findings of this study are available from the corresponding authors.

## Supplementary Materials

The following are available online at https://www.mdpi.com/article/10.3390/healthcare12222285/s1.
