# Supplementary material for: Factors Associated with the Practice of Assessing Drug–Drug Interactions Among Pharmacists in Saudi Arabia
Source: Healthcare (Basel). 2024 Nov 15;12(22):2285. doi: 10.3390/healthcare12222285 (PMC11594466; doi:10.3390/healthcare12222285)
Supplement: Supplementary file 1 [file healthcare-12-02285-s001.zip › healthcare-3211328-supplementary.docx]

**Study Questionnaire**

1. **Demographics**

1. Age in years: ________

2. Gender:

1__Male

2__Female

3. Nationality:

1__Saudi

2__Non-Saudi

4. Marital Status:

1__Married

2__Unmarried

5. Region:

1__Al Jouf

2__The Northern Borders

3__Tabuk

4__Hail

5__Medina

6__Al Qassim

7__Makkah

8__Riyadh

9__Eastern province

10__Al Bahah

11__Asir

12__Jazan

13__Najran

13__Other (Please specify)

6. What is the highest level of education you have completed?

1__Diploma

2__Bachelor’s Degree

3__Pharm D

4__Master’s Degree

5__Other (please specify: _______)

7. When did you earn your highest degree?

1__This year

2__1-5 years ago

3__6-10 years ago

4__11-15 years ago

5__More than 15 years ago

8. Where did you earn your highest degree?

1__Saudi Arabia

2__Egypt

3__Jordan

4__Other (please specify: _____)

9. Where do you currently work?

1__Hospital pharmacy

2__Community pharmacy

3__Other (please specify)

10. How many hours do you work per day?

1__Less than 8 hours/day

2__ 8 hours/day

3__12 hours/day

4__More than 12 hours/day

11. For how long have you been working as a pharmacist?

1__Less than a year

2__1-5 years

3__6-10 years

4__11-15 years

5__More than 15 years

12. What is the type of institution you are working in now?

1__Private

2__Public

**2. Knowledge about DDIs**

13. How do you rate your knowledge about drug-drug interactions?

1__Poor

2__Fair

3__Good

4__Very good

5__Excellent

**14. This section is about the possible drug-drug interactions. To the best of your knowledge, please indicate if there are possible drug interactions of the following drug pairs.**

14.1. Digoxin and sildenafil

1__Should not be used together (contraindicated)

2__May be used together but with monitoring

3__Can be used together (no interaction)

4__Not sure

14.2. Methotrexate and omeprazole

1__Should not be used together (contraindicated)

2__May be used together but with monitoring

3__Can be used together (no interaction)

4__Not sure

14.3. Warfarin and verapamil

1__Should not be used together (contraindicated)

2__May be used together but with monitoring

3__Can be used together (no interaction)

4__Not sure

14.4. Metronidazole and phenytoin

1__Should not be used together (contraindicated)

2__May be used together but with monitoring

3__Can be used together (no interaction)

4__Not sure

14.5. Sildenafil and isosorbide dinitrate

1__Should not be used together (contraindicated)

2__May be used together but with monitoring

3__Can be used together (no interaction)

4__Not sure

14.6. Methotrexate Na and Trimethoprim Sulfamethoxazole

1__Should not be used together (contraindicated)

2__May be used together but with monitoring

3__Can be used together (no interaction)

4__Not sure

14.7. Digoxin and Clarithromycin

1__Should not be used together (contraindicated)

2__May be used together but with monitoring

3__Can be used together (no interaction)

4__Not sure

14.8. Losartan and isosorbide dinitrate

1__Should not be used together (contraindicated)

2__May be used together but with monitoring

3__Can be used together (no interaction)

4__Not sure

14.9. Cyclosporine and Rifampin

1__Should not be used together (contraindicated)

2__May be used together but with monitoring

3__Can be used together (no interaction)

4__Not sure

14.10. Alprazolam and itraconazole

1__Should not be used together (contraindicated)

2__May be used together but with monitoring

3__Can be used together (no interaction)

4__Not sure

14.11. Amiodarone and fluconazole

1__Should not be used together (contraindicated)

2__May be used together but with monitoring

3__Can be used together (no interaction)

4__Not sure

14.12. Warfarin and amiodarone

1__Should not be used together (contraindicated)

2__May be used together but with monitoring

3__Can be used together (no interaction)

4__Not sure

14.13. Aspirin and ibuprofen (occasional use)

1__Should not be used together (contraindicated)

2__May be used together but with monitoring

3__Can be used together (no interaction)

4__Not sure

14.14. Warfarin and atorvastatin

1__Should not be used together (contraindicated)

2__May be used together but with monitoring

3__Can be used together (no interaction)

4__Not sure

14.15. Ferrous fumarate and XR ciprofloxacin

1__Should not be used together (contraindicated)

2__May be used together but with monitoring

3__Can be used together (no interaction)

4__Not sure

14.16. Fexofenadine and metoprolol (succinate tartrate)

1__Should not be used together (contraindicated)

2__May be used together but with monitoring

3__Can be used together (no interaction)

4__Not sure

**3. Attitude Toward DDIs**

**15. Please answer the following questions about drug interactions based on your opinions:**

|  |  | Strongly disagree | Disagree | Neither disagree nor agree | Agree | Strongly agree |
| --- | --- | --- | --- | --- | --- | --- |
| 15.1 | Some drug-drug interactions can be fatal. |  |  |  |  |  |
| 15.2 | Pharmacist should update their knowledge about drug-drug interactions. |  |  |  |  |  |
| 15.3 | Pharmacist should check for drug-drug interactions. |  |  |  |  |  |

**4. Practice Toward DDIs**

16. In general, before dispensing any drug (new or refill), how often do you consider its potential interactions?

1__Never

2__Rarely

3__Sometimes

4__Often

5__Always

17. How often do you ask your patients about their prescription drugs?

1__Never

2__Rarely

3__Sometimes

4__Often

5__Always

18. How often do you ask your patients about their over-the-counter (OTC) drugs?

1__Never

2__Rarely

3__Sometimes

4__Often

5__Always

19. How often do you come across drug-drug interactions?

1__Never

2__Rarely

3__Sometimes

4__Often

5__Always

**5. Pharmacy Characteristics**

20. How would you rate the workload in your pharmacy?

1__Very low

2__Low

3__Moderate

4__High

5__Very high

21.How many pharmacists do you work with during your shift? [Dropdown list]

22.How many technicians do you work with during your shift? [Dropdown list]

23.How many interns do you work with during your shift? [Dropdown list]

24. Does the pharmacy have computer software to check for drug-drug interactions?

1__Yes

2__No

25. When you want to learn more about an interaction, what reference/person do you use? [Select all that apply]

1__Pharmacist

2__Package insert

3__Printed materials (e.g., textbooks)

4__Computerized alert system
5__Internet websites

6__Mobile applications
7__Other

8__None

26. Which of the following mobile applications do you use?

1__Lexicomp

2__Micromedex

3__Medscape

4__Rxlist

5__Other

6__None
